# One-sided anterior Urethroplasty for panurethral stricture: step-by-step

**DOI:** 10.1590/S1677-5538.IBJU.2018.0174

**Published:** 2019-01-29

**Authors:** Willian Eduardo Ito, Marco Aurélio Rodrigues, Silvio Henrique Maia de Almeida

**Affiliations:** 1 Universidade Estadual de Londrina Centro de Ciencias da Saude Londrina PR Brasil Disciplina de Urologia, Universidade Estadual de Londrina Centro de Ciencias da Saude, Londrina, PR, Brasil

## CASE DESCRIPTION

### INTRODUCTION AND OBJECTIVES

The management of complex urethral strictures is surgical challenging, especially for stenosis affecting the entire extension of the anterior urethra.

In this video, we present a step-by-step one-sided anterior urethroplasty for discussion about the surgical aspects of this technique.

### MATERALS AND METHODS

We present a case report of a 23-year-old male patient, complaining of progressive voiding symptoms, bleeding from meatus and perineal pain, which began after a sexual intercourse four months ago. He had no previous urethral surgery, urethral instrumentation or any urethritis treatment. Retrograde urethrography showed a full length stricture of the anterior urethra. Urofluxometry showed a maximum flow of 3mL per second.

We performed the one-sided anterior urethroplasty with oral mucosal graft as described by Kulkarni (1, 2), a minimally invasive technique which preserves the neurovascular supply (3-5).

### RESULTS

The patient’s postoperative recovery was uneventful and the patient had no complain about his graft donor site, with minimal pain, easily managed with common analgesics. On postoperative day one, there was a penile edema, which regressed spontaneously.

After 21 days, the 16Fr Foley catheter was removed and a retrograde urethrography was performed, which has shown a successful improvement of the width of the anterior urethra and a small proximal diverticulum, but the patient referred great subjective urinary flow.

Post-operative uroflowmetry showed a maximum voiding flow of 13mL per second.

### CONCLUSIONS

The Kulkarni’s technique for panurethral strictures is a less invasive and smart technique which spares one side of the urethra neurovascular supply and the operation can be performed in one single stage.
